# School outcomes after HIE: a population-based cohort study

**DOI:** 10.1136/archdischild-2024-328346

**Published:** 2025-06-08

**Authors:** Philippa Rees, Mithilesh Dronavalli, Ben Carter, Barbara Bajuk, Lucinda Burns, Michelle Dickson, John Eastwood, Sadia Hossain, Kate Lawler, Evelyn Lee, Sithum Munasinghe, Andrew Page, Hannah Uebel, Lauren Dicair, Charles Green, Chris Gale, Ju Lee Oei

**Affiliations:** 1 Population Policy and Practice, University College London Institute of Child Health, London, UK; 2 Translational Health Research Institute, Western Sydney University, Penrith, New South Wales, Australia; 3 Department of Biostatistics and Health Informatics, Institute of Psychiatry, Psychology and Neuroscience, King’s College London, London, UK; 4 NSW Pregnancy and Newborn Services Network, Sydney Children’s Hospitals Network, St Pauls, New South Wales, Australia; 5 National Drug and Alcohol Research Centre, University of New South Wales, Randwick, New South Wales, Australia; 6 The Poche Centre for Indigenous Health, The University of Sydney Faculty of Medicine and Health, Sydney, New South Wales, Australia; 7 National Public Health Service, Te Whatu Ora-Health New Zealand, Dunedin, New Zealand; 8 School of Population Health, University of New South Wales, Kensington, New South Wales, Australia; 9 School of Health Sciences, Western Sydney University, Penrith, New South Wales, Australia; 10 Discipline of Paediatrics and Child Health, School of Clinical Medicine, University of New South Wales, Sydney, New South Wales, Australia; 11 Centre for Social Research in Health, University of New South Wales, Sydney, New South Wales, Australia; 12 Centre for Economic Impacts of Genomic Medicine, Macquarie Business School, Macquarie University, Sydney, New South Wales, Australia; 13 Discipline of Paediatrics and Child Health, University of New South Wales, Sydney, New South Wales, Australia; 14 Private Practice, Havertown, Pennsylvania, USA; 15 Alpha Maxx Healthcare, Memphis, Tennessee, USA; 16 Academic Neonatal Medicine, School of Public Health, Imperial College London, London, UK; 17 Department of Newborn Care, Royal Hospital for Women, Randwick, New South Wales, Australia; 18 School of Women's and Children’s Health, University of New South Wales, Randwick, New South Wales, Australia

**Keywords:** Epidemiology, Follow-Up Studies, Neonatology

## Abstract

**Objective:**

To study the school performance of children with hypoxic ischaemic encephalopathy (HIE) relative to population controls from childhood to early adolescence.

**Design:**

Population-based cohort study.

**Setting:**

New South Wales, Australia.

**Patients:**

All 564 159 live-born infants ≥35 weeks’ gestation born between 2008 and 2013 were eligible; 550 with HIE and 558 355 population controls.

**Exposure:**

Mild, moderate-severe HIE.

**Main outcome measures:**

National school assessment performance at 8–9, 10–11 and 12–13 years. Secondary outcomes: reading, writing, spelling, grammar and numeracy scores at these ages. Linear regression models estimated the adjusted mean difference (aMD) at each timepoint. Hierarchical growth-curve modelling assessed academic trajectories (adjusted β).

**Results:**

Children with moderate-severe HIE had significantly lower total z-scores compared with controls at 8–9 years (aMD −0.70; 95% CI −0.84 to –0.52), 10–11 years (aMD −0.96; 95% CI −1.19 to –0.57) and 12–13 years (aMD −0.82; 95% CI −1.12 to –0.53), especially in reading and writing. The gap in overall mean scores remained fixed over time. Despite a lower likelihood of passing each year compared with controls, most infants with moderate-severe HIE passed each year (n=103, 62.4%). Children with mild HIE did not perform significantly differently from controls at 8–9 years (aMD −0.09; 95% CI −0.32 to 0.15), although there was a signal of worsening performance with age (β=13.54; 95% CI –21 to –6.07).

**Conclusions:**

Children with moderate-severe HIE perform at a lower academic level than their peers, yet most meet national standards up to early adolescence. In contrast, children with mild HIE perform on par with their peers. This information is crucial for families: providing reassurance and a basis for needed support.

**WHAT IS ALREADY KNOWN ON THIS TOPIC**

- Children with hypoxic ischaemic encephalopathy (HIE) are at risk for neurodevelopmental issues.

**WHAT THIS STUDY ADDS**

- Children with moderate-severe HIE perform at a lower academic level compared with their peers, but most pass national assessments.
- Children with mild HIE show little difference in academic performance at early school age compared with peers; however, the sample size was small, and larger studies are needed to identify potential subtle later effects.

**HOW THIS STUDY MIGHT AFFECT RESEARCH, PRACTICE OR POLICY**

- Provides robust long-term data to inform counselling of parents in the age of routine therapeutic hypothermia and highlights the need for long-term follow-up and support.
- Indicates that short-term follow-up studies may not adequately detect cognitive effects in mild HIE.

## Introduction

 A diagnosis of hypoxic ischaemic encephalopathy (HIE) is a devastating event for families and affected children, often marking a traumatic end to pregnancy and the start of a difficult journey of uncertainty.^1^ The introduction of an effective treatment, therapeutic hypothermia (TH), has improved outcomes and brought hope to many families. The success of TH in reducing death and disability for infants with moderate-severe HIE, the minimal side effect profile of hypothermia and growing concerns about neurodevelopment following mild HIE have led to a non-evidence-based therapeutic drift such that infants across the severity spectrum of HIE now routinely undergo TH in many high-income countries.^2–4^

TH became the standard of care between 2007 and 2009; therefore, early epochs of infants who received this therapy are only now reaching adulthood. Consequently, little is known about how children’s lives are affected by HIE following TH beyond early childhood, and the developmental trajectories of those with mild HIE are even less well described.

Existing studies have reported that even among survivors of moderate-severe HIE without severe neurodevelopmental impairment, children are more likely to have cognitive and behavioural difficulties.^5–11^ However, these ‘long-term’ follow-up studies focused on early school age, included small populations, or lacked population controls.^5–11^ No previous studies have looked at outcomes beyond early school age relative to healthy controls, despite parents repeatedly highlighting this as a priority question for families.^12 13^ Multiple studies have also demonstrated the poor ability of early childhood outcomes to predict later outcomes.^14–19^

To address these gaps, we conducted a cohort study to explore the school performance of children with mild, moderate and severe HIE in the TH era, compared with peers without HIE.

## Methods

We conducted a population-based prospective cohort study of infants born and diagnosed with HIE in New South Wales (NSW), Australia between 2008 and 2013. The study’s aim was to determine the impact of HIE (mild and moderate-severe) on school performance relative to population controls from childhood to early adolescence.

### Cohort creation

The Centre for Health Record Linkage linked the data of all infants born in NSW across multiple datasets, using probabilistic matching of personal identifiers (table 1).^20^ The HIE cohort for this study was created using the Neonatal Intensive Care Units (NICUs) dataset (capturing data from all NSW NICUs), and the control cohort was identified from the Perinatal Data Collection (PDC). These were also linked to the Admitted Patient Data Collection (APDC), the Australian Bureau of Statistics Cause of Death, the Cerebral Palsy Register and the National Assessment Programme Literacy and Numeracy (NAPLAN) database to ascertain covariates and outcomes (table 1).

**Table 1:** Databases used in this cohort study

NICUs data collection	Contains data on all eligible infants admitted to a NICU in New South Wales in the first 28 days of life. Eligibility criteria include infants who have a central line, undergo therapeutic hypothermia, require assisted ventilation (mechanical ventilation, continuous positive pressure or high flow oxygen or air at >1 L/min), receive major surgery, receive an exchange transfusion and preterm (<32 weeks’ gestation) or low birth weight (≤1500 g) infants. Data are demographic, diagnostic and related to treatment for all reported infants.^31^
New South Wales Perinatal Data Collection	Contains maternal and infant birth data for every infant born in New South Wales. This dataset was used to identify which of the infants in the NICUs dataset were born in New South Wales and eligible for inclusion.^20^
APDC	Contains data on all hospital admissions (discharges, transfers and deaths) for all New South Wales residents. It was used to identify infants who had died and to retrieve additional covariates for the cohort.^20^
Australian Bureau of Statistics Cause of Death	Details the cause of death for all New South Wales residents. These data were corroborated with the APDC dataset to exclude infants who had died.^20^
Cerebral Palsy Registry	Contains demographic data, details on the type of cerebral palsy and other medical information.^20^
NAPLAN database	Contains data on child and parental characteristics and data related to the standardised NAPLAN testing at grade 3 (age 8–9 years), 5 (10–11 years), 7 (12–13 years) and 9 (14–15 years).^32 33^ The NAPLAN is a nationwide centralised assessment that was introduced in 2008 and is compulsory for all Australian schools. It allows benchmarking and tracking of individual pupils’ performance over time, relative to their school peers and relative to their peers nationwide. It does not replace school or teacher-based assessments. The NAPLAN tests across five domains: reading, writing, spelling, grammar and numeracy. Each test is scored 0–1000 and the results are scaled across grades so that scores are equivalent across grades. There is a predetermined NMS for each grade of the NAPLAN. Few children are exempt from the NAPLAN tests, but those that do not sit the NAPLAN are considered to not meet the NMS.

APDC, Admitted Patient Data Collection; NAPLAN, National Assessment Programme Literacy and Numeracy; NICUs, neonatal intensive care units; NMS, National Minimum Standard.

### Participants

Infants reported in the NSW NICUs dataset born between 2008 and 2013 at ≥35 weeks’ gestation and given a diagnosis of HIE were included. Infants were categorised as having either moderate-severe HIE or mild HIE (using the worst grade of HIE). NSW eligibility criteria for TH included infants ≥35 weeks’ gestation, ≥1800 g, within 6 hours of birth; with severe perinatal acidosis or hypoxia; and signs of moderate-severe encephalopathy or seizures (clinical or electrographic).^21^ TH for mild HIE was considered at the clinician’s discretion.^21^ Nine NICUs offered TH across NSW.

Infants diagnosed with mild HIE but with documented seizures on the neonatal unit were reclassified as moderate HIE by PR. Population controls included all infants born in NSW between 2008 and 2013 with the same gestational age restrictions but who were not included in the NICUs dataset.

### Covariates

Key covariates such as sex, maternal smoking, indigenous status, parental education, Accessibility/Remoteness Indicator of Australia (ARIA) score, birth year, week of gestation, birth weight z-score and socioeconomic status (SES) decile 2011 were derived from PDC or APDC records. Parental education status was available in the NAPLAN dataset.

### Outcomes

Primary outcome:

- School performance: Total z-score across the five domains assessed at grades 3, 5 and 7.

Secondary outcomes in grades 3, 5 and 7:

- Reading z-score.
- Writing z-score
- Spelling z-score.
- Grammar z-score.
- Numeracy z-score.
- Meeting the National Minimum Standard (NMS) overall.

Meeting the NMS in:

- Reading.
- Writing
- Spelling.
- Grammar.
- Numeracy.

### Data analysis

All surviving participants with a NAPLAN record were included. The characteristics of infants with moderate-severe HIE, mild HIE and controls were described.

Total z-scores were created across the five assessed domains at grades 3, 5 and 7 for each included infant, in addition to z-scores for the individual domains.^22^ Crude and adjusted mean difference (aMD) in z-scores for children were compared for infants with moderate-severe HIE and mild HIE relative to controls, using linear regression. An aMD of 0.2 SDs was previously determined to be functionally significant.^22^ The odds of meeting the NMS at each grade and for each domain were determined using logistic regression. Analyses were adjusted for prespecified confounders.

Hierarchical growth-curve modelling was undertaken to delineate the trajectories of mean academic scores over time for each group. This was done using a linear mixed effects regression, fitting baby as a random effect, enabling the inclusion of all children and accounting for those missing in later years. This technique allows for the estimation of two parameters: the intercept, representing initial academic performance at grade 3, and the slope (β), reflecting the rate of change in scores from grade 3 to 7. By incorporating a group-by-time interaction term, the model captures differential slopes associated with HIE severity while accounting for confounders. Analyses were conducted in Stata V.18.

Children with no attainment score who did not meet the NMS were assigned the lowest score for their test year.^22^ Missing NAPLAN outcome data were predominantly due to the cancellation of school assessments in 2020 due to COVID-19. Given that this missingness was not random and likely to affect school performance, a complete case analysis was undertaken. Outside of the pandemic, the missingness of NAPLAN data was low: 79%–88% of included children had corresponding NSW NAPLAN records. Outside of 2020, those with missing NAPLAN records may have moved out of state, as we only had access to NSW records. Outcomes of survivors of HIE were compared by TH status in subgroup analyses.

## Results

Between 2008 and 2013, 550 infants with HIE were identified: 124 with mild HIE (22.5%), 386 with moderate-severe HIE (70.2%) and 40 with unspecified severity (7.3%; online supplemental file 1). Infants with HIE had similar characteristics to controls; the mean gestational age was 39.2 (SD=1.5; table 2).

**Table 2:** Characteristics of population surviving until school age and included in National Assessment Programme Literacy and Numeracy analysis

Characteristic	Moderate-severe HIE (n=165) mean (SD)	Mild HIE (n=66) mean (SD)	Controls n=340 975 mean (SD)
Gestation (weeks)	39.1 (1.3)	39.2 (1.6)	39.1 (1.3)
Birth weight (g)	3315 (597)	3417 (592)	3424 (496)
Birth weight z-score (WHO chart)	−0.02 (1.21)	0.16 (1.09)	0.24 (0.97)
Apgar at 5 min	4.6 (2.6)	4.4 (2)	9 (0.6)
Socioeconomic status decile 2011	5.1 (2.8)	5.4 (2.8)	5.2 (2.8)
Accessibility/Remoteness Indicator of Australia score	10.2 (0.6)	10.2 (0.6)	10.3 (0.6)

	Moderate-severe HIE n (%)	Mild HIE n (%)	Controls, n (%)
Maternal smoking	22 (13.3)	10 (15.2)	37 482 (11)
Male infant	84 (50.91)	43 (65.15)	172 281 (50.53)
Mode of delivery			
Spontaneous vaginal delivery	58 (35.12)	20 (30.3)	199 349 (58.46)
Instrumental vaginal delivery	34 (20.61)	16 (24.24)	38 722 (11.36)
Caesarean section	73 (44.24)	30 (45.45)	102 853 (30.16)
Unknown	0 (0)	0	51 (0.01)
Indigenous status	13 (7.9)	7 (10.6)	22 621 (6.64)
Educated parents	129 (82.69)	61 (95.31)	284 296 (87.75)

HIE, hypoxic ischaemic encephalopathy.

Most infants with moderate-severe HIE had neonatal seizures (n=308, 79.8%) and received mechanical ventilation (n=317, 82.1%) and anticonvulsant therapy during their neonatal unit stay (n=305, 79%). Inotropes were administered to 39.9% (n=154) of infants with moderate-severe HIE and 21% (n=26) with mild HIE. Suspected or proven chorioamnionitis was documented for 8.8% (n=24) with moderate-severe HIE and 8.1% (n=10) with mild HIE. Most infants with moderate-severe HIE were born in non-tertiary centres (n=255, 66.1%), whereas most included infants with mild HIE were born in tertiary centres (n=64, 51.6%).

Of those with moderate-severe HIE, 67.1% (n=259) underwent TH for a median of 72 hours. Similarly, most of those with mild HIE also underwent TH (n=76, 61.2%) for a median of 70.5 hours. Between 2008 and 2013 receipt of TH for those with moderate-severe HIE increased from 49.2% to 77.1% and from 26.3% to 91.3% in those with mild HIE. Infants with moderate-severe HIE who did not receive TH had double the mortality rate (n=52, 40.9% vs n=58, 22.4%). Infants who received TH were also admitted to the neonatal unit earlier (median time to the first line, 5.1 hours vs 8.7 hours). However, the ARIA (rurality) score was similar for those who did and did not receive TH.

Of these infants, 249 surviving children with HIE and NAPLAN data for analysis were included alongside 340 975 controls (online supplemental file 1). Outcome completeness was similar for cases (84.1%) and controls (87%) (pre-COVID NAPLAN figures).

### School performance after moderate-severe HIE

#### Grade 3

At 8–9 years, 165 survivors of moderate-severe HIE underwent the NAPLAN assessment, 28 with severe and 137 with moderate HIE. Most (n=118, 71.5%) received TH, and 24.2% (n=40) had cerebral palsy.

Children with moderate-severe HIE had significantly lower total z-scores and lower z-scores for each of the five domains assessed compared with controls (total aMD −0.70; 95% CI −0.84 to –0.52) (table 3). This effect was largest for reading (aMD −0.69; 95% CI −0.84 to –0.54), writing (aMD −0.67; 95% CI −0.82 to –0.53) and numeracy (aMD −0.70; 95% CI −0.85 to –0.55) (table 3). Although children with moderate-severe HIE were less likely to pass the grade 3 assessment (and to pass each domain) compared with controls (aOR 0.4; 95% CI 0.3 to 0.5), most did pass (n=112, 67.9%). These effects were seen for those who did and did not receive TH, although those who did not receive TH had lower scores and were less likely to pass (online supplemental file 2).

**Table 3:** School performance of infants with moderate-severe HIE compared with controls

Grade 3	Moderate to severe HIE (n=165)	Controls (n=338, 027)	Crude mean difference (95% CI)	Adjusted mean difference (95% CI)
Total z-score across five domains (SD)	−0.77 (1.5)	0 (1)	−0.77 (–0.92 to –0.61)	−0.70 (–0.84 to –0.52)
Reading z-score (SD)	−0.76 (1.5)	0 (1)	−0.76 (–0.91 to –0.61)	−0.69 (–0.84 to –0.54)
Writing z-score (SD)	−0.74 (1.6)	0 (1)	−0.74 (–0.90 to –0.59)	−0.67 (–0.82 to –0.53)
Spelling z-score (SD)	−0.50 (1.2)	0 (1)	−0.50 (–0.66 to –0.35)	−0.45 (–0.60 to –0.30)
Grammar z-score (SD)	−0.65 (1.4)	0 (1)	−0.65 (–0.80 to –0.49)	−0.58 (–0.73 to –0.43)
Numeracy z-score (SD)	−0.75 (1.5)	0 (1)	−0.75 (–0.91 to –0.60)	−0.70 (–0.85 to –0.55)
**Met National minimum standard**	**n (%)**	**n (%)**	**OR (95% CI)**	**aOR (95% CI)**
Reading pass	123 (74.6)	316 313 (93.6)	0.2 (0.1 to 0.3)	0.2 (0.1 to 0.3)
Writing pass	129 (78.2)	319 322 (94.5)	0.2 (0.2 to 0.3)	0.2 (0.2 to 0.3)
Spelling pass	127 (77)	313 436 (92.7)	0.3 (0.2 to 0.4)	0.3 (0.2 to 0.4)
Grammar pass	128 (77.6)	312 108 (92.3)	0.3 (0.2 to 0.4)	0.3 (0.2 to 0.5)
Numeracy pass	121 (73.3)	316 332 (93.6)	0.2 (0.1 to 0.3)	0.2 (0.1 to 0.3)
Total pass all domains	112 (67.9)	291 378 (86.2)	0.3 (0.2 to 0.5)	0.4 (0.3 to 0.5)

Grade 5	Moderate-severe HIE (n=74)	Controls (n=177 843)	Crude mean difference (95% CI)	Adjusted mean difference (95% CI)
Total z-score across five domains (SD)	−1.04 (1.85)	0 (1)	−1.04 (–1.28 to –0.82)	−0.96 (–1.19 to –0.57)
Reading z-score (SD)	−1.04 (1.9)	0 (1)	−1.04 (–1.26 to –0.8)	−0.93 (–1.15 to –0.72)
Writing z-score (SD)	−0.98 (1.8)	0 (1)	−0.98 (–1.2 to –0.75)	−0.91 (–1.13 to –0.70)
Spelling z-score (SD)	−0.72 (1.5)	0 (1)	−0.72 (–0.94 to –0.49)	−0.66 (–0.88 to –0.44)
Grammar z-score (SD)	−0.96 (1.7)	0 (1)	−0.96 (–1.19 to –0.73)	−0.89 (–1.1 to –0.67)
Numeracy z-score (SD)	−0.91 (1.6)	0 (1)	−0.91 (–1.14 to –0.68)	−0.83 (–1.05 to –0.62)
**Met National minimum standard**	**n (%)**	**n (%)**	**OR (95% CI)**	**aOR (95% CI)**
Reading pass	54 (73)	166 032 (93.4)	0.2 (0.1 to 0.3)	0.2 (0.1 to 0.4)
Writing pass	51 (68.9)	163 254 (91.8)	0.2 (0.1 to 0.3)	0.2 (0.1 to 0.3)
Spelling pass	53 (71.6)	165 244 (92.9)	0.2 (0.1 to 0.3)	0.2 (0.1 to 0.3)
Grammar pass	47 (63.5)	162 723 (91.5)	0.2 (0.1 to 0.3)	0.2 (0.1 to 0.3)
Numeracy pass	52 (70.3)	166 763 (93.8)	0.2 (0.1 to 0.3)	0.2 (0.1 to 0.3)
Total pass all domains	44 (59.5)	150 605 (84.7)	0.3 (0.2 to 0.4)	0.3 (0.2 to 0.5)

Grade 7	Moderate- severe HIE (n=38)	Controls (n=75 163)	Crude mean difference (95% CI)	Adjusted mean difference (95% CI)
Total z-score across five domains (SD)	−0.84 (1.7)	0 (1)	−0.84 (–1.16 to –0.52)	−0.82 (–1.12 to –0.53)
Reading z-score (SD)	−0.84 (1.6)	0 (1)	−0.84 (–1.11 to –0.52)	−0.82 (–1.12 to –0.52)
Writing z-score (SD)	−0.77 (1.6)	0 (1)	−0.77 (–1.09 to –0.46)	−0.77 (–1.06 to –0.47)
Spelling z-score (SD)	−0.51 (1.2)	0 (1)	−0.51 (–0.83 to –0.19)	−0.48 (–0.79 to –0.18)
Grammar z-score (SD)	−0.78 (1.6)	0 (1)	−0.78 (–1.09 to –0.46)	−0.77 (–1.08 to –0.47)
Numeracy z-score (SD)	−0.7 (1.4)	0 (1)	−0.7 (–1.02 to –0.38)	−0.68 (–0.98 to –0.38)
**Met National minimum standard**	**n (%**)	**n (%)**	**OR (95% CI)**	**aOR (95% CI)**
Reading pass	28 (73.7)	69 073 (91.9)	0.3 (0.1 to 0.5)	0.2 (0.1 to 0.5)
Writing pass	28 (73.7)	67 603 (89.9)	0.3 (0.2 to 0.6)	0.3 (0.1 to 0.7)
Spelling pass	28 (73.7)	68 934 (91.7)	0.3 (0.1 to 0.5)	0.2 (0.1 to 0.5)
Grammar pass	29 (76.3)	66 348 (88.3)	0.4 (0.2 to 0.9)	0.5 (0.2 to 1.1)
Numeracy pass	28 (73.7)	68 332 (90.9)	0.3 (0.1 to 0.6)	0.3 (0.1 to 0.6)
Total pass all domains	27 (71.05)	59 998 (79.8)	0.6 (0.3 to 1.3)	0.6 (0.3 to 1.4)

Adjusted for sex, maternal smoking, Indigenous status, parental education, Accessibility/Remoteness Indicator of Australia score, birthyear, gestation, birth weight Z score, socioeconomic status decile 2011.

aOR, adjusted OR; HIE, hypoxic ischaemic encephalopathy.

#### Grade 5

At 10–11 years, most survivors of moderate-severe HIE had undergone TH (n=45, 60.8%). These children had significantly lower total z-scores than their peers overall (aMD −0.96; 95% CI −1.19 to –0.75) and for each domain—particularly reading and writing (table 3). They were also less likely to pass grade 5 (aOR 0.3; 95% CI 0.2 to 0.5) in each of the domains assessed compared with their peers. However, most of them did pass the national assessment (n=45, 60%), although fewer than observed at grade 3 (n=112, 67.9%) (table 3). These effects were also seen in subgroup analyses including only those who received TH (online supplemental file 2).

#### Grade 7

At 12–13 years, around half of survivors of moderate-severe HIE had undergone TH (n=17, 44.7%). Significantly lower total z-scores persisted for this group at 12–13 years (aMD −0.82; 95% CI −1.12 to –0.53): this was observed for all five domains (table 3). Scores remained particularly low for reading and writing.

**Figure 1:**
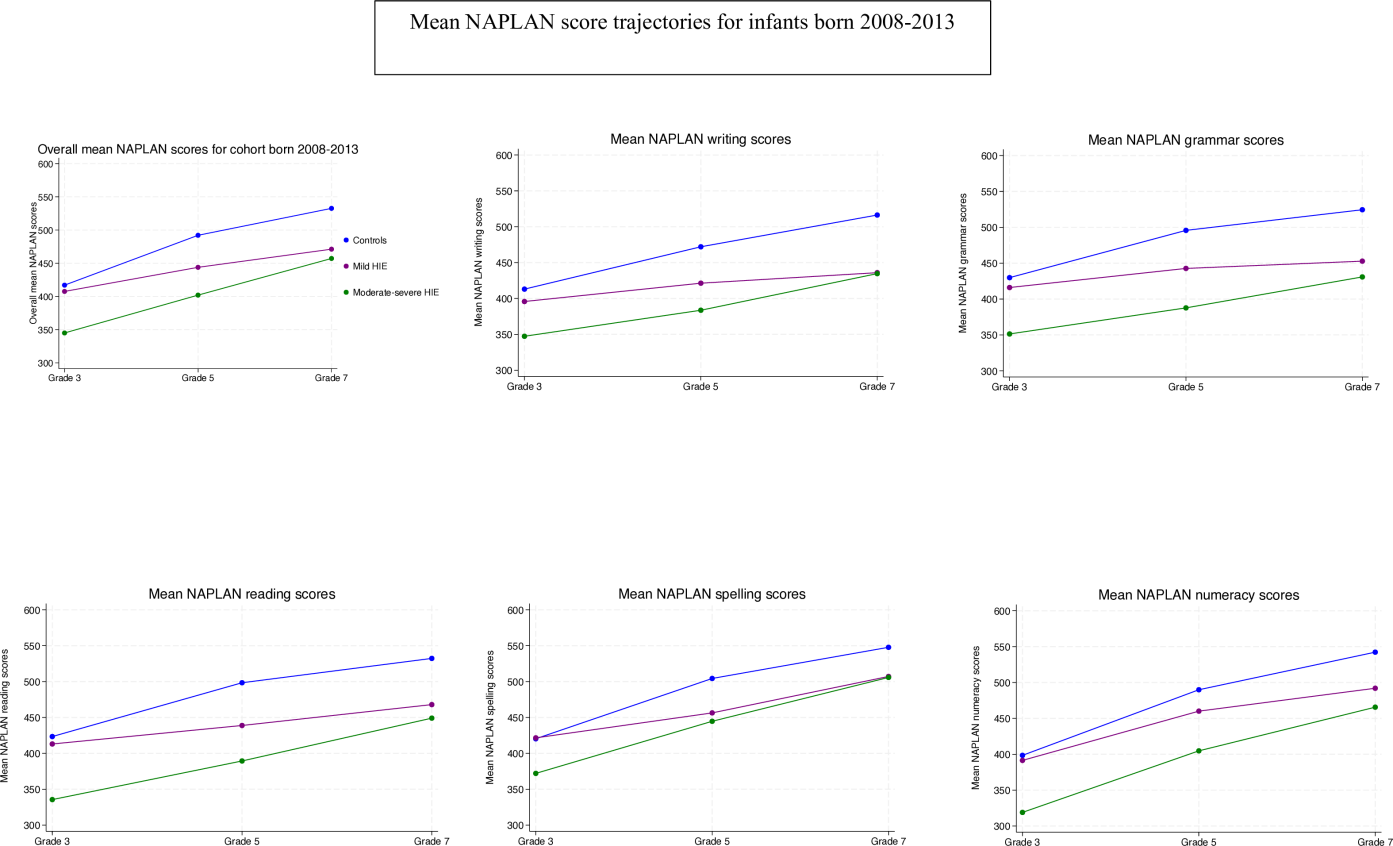
Mean overall and domain-specific academic score trajectorires for infants with moderate-severe HIE, mild HIE and controls born between 2008-2013.

Children with moderate-severe HIE were less likely to pass most of the assessed domains compared with controls (except for grammar, for which pass rates were comparable) (table 3). Most children with moderate-severe HIE, however, did pass each domain and the overall assessment (n=27, 71.1%). The mean scores of those with moderate-severe HIE were lower than controls at this age (figure 1), but there was no significant difference in their chance of passing the assessment year (aOR 0.6; 95% CI 0.3 to 1.4) (although the number of children included in this age bracket was low; table 3).

#### Growth modelling of academic trajectories

NAPLAN mean scores showed a similar upward trend for children with moderate-severe HIE compared with controls from grades 3 to 7 (figure 1). This pattern was consistent across all domains (figure 1). However, children with moderate-severe HIE had significantly lower scores than controls throughout their school years (intercept aMD −57.2; 95% CI −78.6 to –35.7), and this persisted after limiting analyses to those who received TH (intercept aMD −37.4; 95% CI –64.9 to –10) (online supplemental file 3–5). Notably, the gap in aMD throughout school years remained fixed relative to controls (adjusted β=−3.5; 95% CI −8 to 1).

### Mild HIE

#### Grade 3

More than half of the children with mild HIE included in the grade 3 NAPLAN assessment (age 8–9 years) had undergone TH (n=39, 60%). Children with mild HIE (n=65) had similar attainment scores at 8–9 years of age compared with controls overall (aMD −0.09; 95% CI −0.32 to 0.15) and for each domain (table 4). There was also no difference in their likelihood of passing the grade 3 assessment relative to controls (aOR 1; 95% CI 0.5 to 2.1).

**Table 4:** School performance of infants with mild HIE compared with controls

Grade 3	Mild HIE (n=65)	Controls (n=338 027)	Crude mean difference (95% CI)	Adjusted mean difference (95% CI)
Total z-score across five domains (SD)	−0.1 (1.3)	0 (1)	−0.1 (–0.34 to 0.14)	−0.09 (–0.32 to 0.15)
Reading z-score (SD)	−0.09 (1.2)	0 (1)	−0.09 (–0.33 to 0.15)	−0.07 (–0.31 to 0.16)
Writing z-score (SD)	−0.19 (1.3)	0 (1)	−0.19 (–0.44 to 0.05)	−0.17 (–0.4 to 0.06)
Spelling z-score (SD)	0.01 (1.1)	0 (1)	0.01 (–0.23 to 0.26)	0.02 (–0.22 to 0.25)
Grammar z-score (SD)	−0.1 (1.2)	0 (1)	−0.1 (–0.36 to 0.13)	−0.09 (–0.32 to 1.5)
Numeracy z-score (SD)	−0.07 (1.2)	0 (1)	−0.07 (–0.31 to 0.18)	−0.07 (–0.31 to 0.16)
**Met National minimum standard**	**n (%**)	**n (%)**	**OR (95% CI**)	**aOR (95% CI**)
Reading Pass	58 (89.2)	316 313 (93.6)	0.6 (0.3 to 1.3)	0.6 (0.3 to 1.2)
Writing Pass	58 (89.2)	319 322 (94.5)	0.5 (0.2, to 1.1)	0.5 (0.2 to 1.1)
Spelling Pass	57 (87.7)	313 436 (92.7)	0.6 (0.3 to 1.2)	0.6 (0.3 to 1.2)
Grammar Pass	57 (87.8)	312 108 (92.3)	0.6 (0.3 to 1.2)	0.7 (0.3 to 1.5)
Numeracy Pass	58 (89.2)	316 322 (93.6)	0.6 (0.3 to 1.2)	0.7 (0.3 to 1.5)
Total Pass all domains	55 (84.6)	291 378 (86.2)	0.9 (0.4 to 1.7)	1 (0.5 to 2.1)

Grade 5	Mild HIE group (n=28)	Controls n=177 843	Crude Mean difference (95% CI)	Adjusted mean difference (95% CI)
Total z-score across five domains (SD)	−0.56 (1.5)	0 (1)	−0.56 (–0.93 to –0.19)	−0.60 (–0.95 to –0.25)
Reading z-score (SD)	−0.57 (1.7)	0 (1)	−0.57 (–0.94 to –0.2)	−0.61 (–0.97 to –0.26)
Writing z-score (SD)	−0.56 (1.5)	0 (1)	−0.56 (–0.93 to –0.19)	−0.57 (–0.92 to –0.22)
Spelling z-score (SD)	−0.58 (1.1)	0 (1)	−0.58 (–0.95 to –0.21)	−0.59 (–0.94 to –0.23)
Grammar z-score (SD)	−0.47 (1.4)	0 (1)	−0.47 (–0.84 to –0.1)	−0.51 (–0.86 to –0.15)
Numeracy z-score (SD)	−0.32 (1.4)	0 (1)	−0.32 (–0.69 to 0.05)	−0.38 (–0.74 to –0.02)
**Met National minimum standard**	**n (%)**	**n (%)**	**OR (95% CI)**	**aOR (95% CI)**
Reading pass	23 (82.1)	166 032 (93.4)	0.3 (0.1, 0.9)	0.3 (0.1 to 0.8)
Writing pass	23 (82.4)	163 254 (91.8)	0.4 (0.2 to 1.1)	0.4 (0.1 to 1)
Spelling pass	24 (85.7)	165, 244 (92.9)	0.5 (0.2 to 1.3)	0.4 (0.1 to 1.2)
Grammar pass	24 (85.7)	162, 723 (91.5)	0.6 (0.2 to 1.6)	0.5 (0.2 to 1.4)
Numeracy pass	24 (85.7)	166 763 (93.8)	0.4 (0.1 to 1.1)	0.3 (0.1 to 1)
Total pass all domains	22 (78.6)	150 605 (84.7)	0.7 (0.3 to 1.6)	0.6 (0.2 to 1.5)

Grade 7	Mild HIE (n=13)	Controls (n=75 163)	Crude mean difference (95% CI)	Adjusted mean difference (95% CI)
Total z-score across five domains (SD)	−0.68 (1.6)	0 (1)	−0.68 (–1.23 to –0.14)	−0.71 (–1.21 to –0.21)
Reading z-score (SD)	−0.65 (1.5)	0 (1)	−0.65 (–1.19 to –0.1)	−0.68 (–1.18 to –0.17)
Writing z-score (SD)	−0.76 (1.7)	0 (1)	−0.76 (–1.3 to –0.22)	−0.77 (–1.28 to –0.26)
Spelling z-score (SD)	−0.49 (1.1)	0 (1)	−0.49 (–1.04 to 0.05)	−0.48 (–1 to 0.03)
Grammar z-score (SD)	−0.59 (1.5)	0 (1)	−0.59 (–1.14 to –0.05)	−0.61 (–1.12 to –0.1)
Numeracy z-score (SD)	−0.46 (1.3)	0 (1)	−0.46 (–1 to 0.08)	−0.49 (–1 to 0.02)
**Met National Minimum Standard**	**n (%)**	**n (%)**	**OR (95% CI)**	**aOR (95% CI)**
Reading pass	10 (76.9)	69 073 (91.9)	0.3 (0.1 to 1.1)	0.2 (0.1 to 0.9)
Writing pass	10 (76.9)	68 6032 (89.9)	0.4 (0.1 to 1.4)	0.3 (0.1 to 1.2)
Spelling pass	10 (76.9)	68 934 (91.7)	0.3 (0.1 to 1.1)	0.3 (0.1 to 1)
Grammar pass	10 (76.9)	66 348 (88.3)	0.4 (0.1 to 1.6)	0.4 (0.1 to 1.4)
Numeracy pass	11 (84.6)	68 332 (90.9)	0.5 (0.1 to 2.5)	0.5 (0.1 to 2.3)
Total pass all domains	10 (76.9)	59 998 (79.8)	0.8 (0.2 to 3.1)	0.8 (0.2 to 3.1)

Adjusted for sex, maternal smoking, Indigenous status, parental education, Accessibility/Remoteness Indicator of Australia score, birthyear, gestation, birth weight Z score, socioeconomic status decile 2011.

aOR, adjusted OR; HIE, hypoxic ischaemic encephalopathy.

#### Grade 5

Only 28 infants with mild HIE had grade 5 educational outcomes available, 10 of which (35.7%) had undergone TH. Despite the low numbers, there appeared to be a possible association between mild HIE and lower overall z-scores at 10–11 years compared with controls (aMD −0.6; 95% CI −0.95 to –0.25; table 4). This effect was largest in the spelling domain (aMD −0.59; 95% CI −0.94 to –0.23). Children with mild HIE were less likely to pass the reading (aOR 0.3; 95% CI 0.1 to 0.8), writing (aOR 0.4; 95% CI 0.1 to 1) and numeracy (aOR 0.3; 95% CI 0.1 to 1) domains relative to controls. However, most did pass these domains and the overall assessment (n=22, 78.6%). There was no significant difference in overall pass rates (aOR 0.6; 95% CI 0.2 to 1.5), but caution is needed due to the underpowered nature of the finding.

#### Grade 7

Few included infants with mild HIE had outcome data available at 12–13 years (n=13). These results are therefore uncertain and should be interpreted with caution (table 4). A difference in the overall z-scores was observed for those with mild HIE relative to controls (aMD 0.7; 95% CI −1.2 to –0.2). There was, however, no difference in the overall pass rates (aOR 0.8; 95% CI 0.2 to 3.1; table 4).

#### Growth modelling of academic trajectories

Infants with mild HIE did not have significantly different scores compared with controls between grades 3 and 7 (intercept aMD −8.1; 95% CI −29.2 to 13). However, those with mild HIE performed worse compared with controls with increasing age (adjusted β=−13.54; 95% CI –21 to –6.07) (online supplemental file 1). Caution should be taken when interpreting this finding due to the low number of mild HIE cases.

## Discussion

This is the first population-based cohort study to report on the school outcomes of children with mild, moderate and severe HIE to adolescence after the adoption of TH as standard of care compared with controls. Children with moderate-severe HIE had consistently lower academic scores relative to controls throughout childhood. However, promisingly, most surviving children with moderate-severe HIE passed the NAPLAN despite lower scores. Reassuringly, children with mild HIE had similar attainment to controls at early school age. There was a suggestion of poorer performance in later school years, although this should be interpreted with caution given the small sample size.

### Strengths and limitations

The use of state-level linked routine data enabled the inclusion of regionally representative participants, reducing selection bias. This approach also facilitated the identification and adjustment of potential confounders. Furthermore, the use of national standardised educational assessments provided a crucial and meaningful measure of how infants with HIE perform relative to their peers but also enabled standardised longitudinal follow-up and exploration of academic trajectories.

This study did, however, have several limitations. First, the outcomes of infants born between 2008 and 2013 might not accurately represent the outcomes of infants born with HIE today. While TH was the standard of care during this period, its administration—particularly time to reach target temperature—and subsequent outcomes have shown improvement in recent years.^1 23–25^ We were also limited by the data available: we did not have detailed information on eligibility for TH or time to initiation. The administration of TH was lower in the earlier years of the study, likely indicating incomplete uptake of this then new treatment, which could influence the results. The birth year was, however, included as a covariate in adjusted analyses, and subgroup analyses limited to infants who received TH showed comparable results. The analysis of longer-term outcomes, particularly for those with mild HIE, was constrained by small sample sizes.

Neonatal units in the NICUs dataset must register infants meeting specific criteria, such as those receiving TH. While most infants with HIE are likely included, milder cases may be underrepresented. This selection bias could skew outcomes for the broader population of infants with mild HIE, affecting the generalisability of findings.

### Context of current literature and implications for policy and practice

The finding of poorer cognitive scores at an early school age after moderate-severe HIE aligns with previous studies. At 6–7 years, the NICHD and TOBY cooling trials (which included 97 and 145 surviving infants, respectively) reported that 27% had intelligent quotient (IQ) scores below 70 and that 52% had an IQ of 85 or greater.^5 11^ A contemporary UK cohort of infants with moderate-severe HIE but without cerebral palsy (n=29) also reported lower IQ scores at 6–8 years of age, alongside lower perceptual reasoning, working memory, processing speed and verbal comprehension.^10 26^

Although Grossmann *et al* reported normal IQ scores among those with moderate-severe HIE at 6–7 years of age (across 46 infants), they did note a trend towards emerging cognitive deficits in later childhood (by 10–12 years).^9^ Our study, however, showed a fixed rather than growing deficit in academic performance with increasing age. This may be due to differences in the HIE population (characteristics or size) or in outcome assessment. However, reassuringly, we demonstrate that despite identifiable deficits on formal testing, most children with moderate-severe HIE pass their school assessments and continue to progress academically. This finding is crucial information to share with families. It also offers valuable guidance on where to focus support efforts, such as on reading and writing, as children grow older. Sharing this key information with parents, children and schools is essential. Furthermore, it underscores the importance of long-term follow-up and support for this population, alongside highlighting the limitations of short-term study outcomes.

Few studies report cognitive or school-age outcomes after mild HIE. Although some small studies indicate lower cognitive scores in early childhood (ages 2 and 5) following mild HIE, we did not observe a significant impact on school performance.^27–30^ This discrepancy might be due to the difficulty in accurately measuring cognitive performance in early childhood, poor translation of early cognitive scores into functional abilities, the small and often heterogeneous samples of previous studies or the possibility that functional cognitive impacts following mild HIE emerge later in childhood.^29^ These considerations underscore the need for adequately powered long-term follow-up studies of infants with mild HIE, as the current understanding of the impact of the initial insult on subsequent development is limited. Additionally, this study suggests that the required sample sizes for adequately powered trials in this population may be larger than currently estimated and that 2-year study endpoints may be insufficient if cognitive sequelae are not observed until later childhood.

## Conclusion

This study reports that individuals with moderate-severe HIE have lower academic attainment. However, it is encouraging to note that these children continue to progress academically, with similar trajectories to their peers over time, and that most pass their assessments successfully. This is valuable information for families. To better understand long-term outcomes following HIE, especially mild HIE, further large-scale, longitudinal studies are crucial, as some effects might not become apparent until later in childhood.

## Supplementary material

10.1136/archdischild-2024-328346 online supplemental file 1

## Data Availability

No data are available.
