# Supplementary material for: School outcomes after HIE: a population-based cohort study
Source: Arch Dis Child Fetal Neonatal Ed. 2025 Jun 8;111(4):e328346. doi: 10.1136/archdischild-2024-328346 (PMC13479228; doi:10.1136/archdischild-2024-328346)
Supplement: Supplementary file 1 [file fetalneonatal-111-4-s001.pdf]

Supplement 1: flow diagram

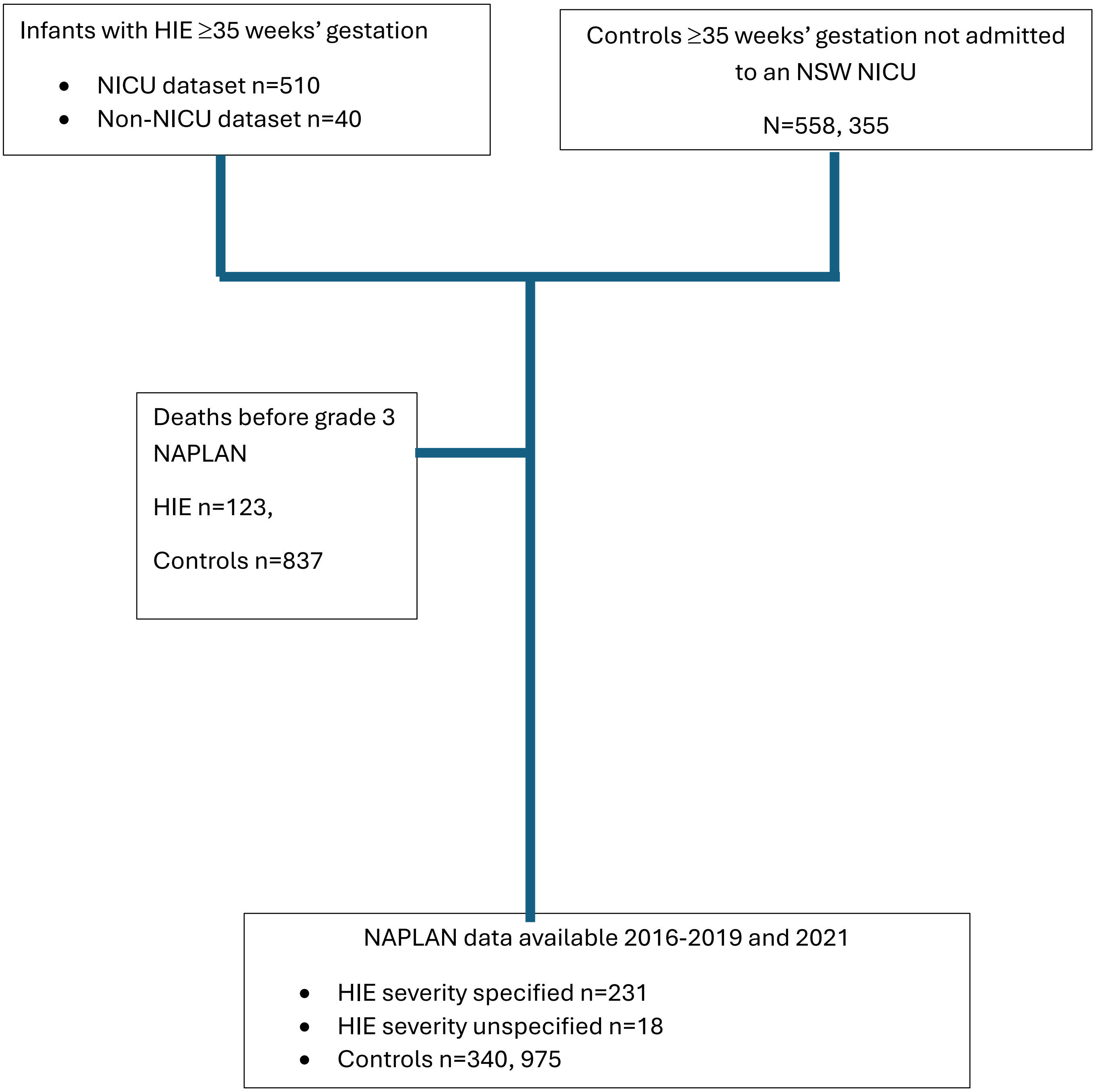

**Supplement 2:** Sub-group analyses of school performance of children with moderate-severe HIE relative to controls stratified by receipt of therapeutic hypothermia				
**Grade 3**				
**Outcome**	**Therapeutic hypothermia (n=118)**	**Controls (n=338, 027)**	**Crude mean difference (95% CI)**	**Adjusted mean difference (95% CI)**
Total z-score across 5 domains (SD)	-0.59 (1.4)	0 (1.0)	-0.59 (-0.77, -0.41)	-0.51 (-0.69, -0.34)
	**Therapeutic hypothermia (n=118)**	**Controls (n=338, 027)**	**OR (95% CI)**	**aOR (95% CI)**
Met National Minimum Standard n (%)	85 (72.0)	291, 378 (86.2)	0.41 (0.28, 0.62)	0.46 (0.29, 0.72)
	**No therapeutic hypothermia (n=47)**	**Controls N=338, 027**	**Crude mean difference (95% CI)**	**Adjusted mean difference (95% CI)**
Total z-score across 5 domains (SD)	-1.21 (1.7)	0 (1.0)	-1.21 (-1.50, -0.93)	-1.13 (-1.40, -0.86)
	**No therapeutic hypothermia (n=47)**	**Controls N=338, 027**	**OR (95% CI)**	**aOR (95% CI)**
Met National Minimum Standard n (%)	27 (57.5)	291, 378 (86.2)	0.22 (0.12, 0.39)	0.21 (0.11, 0.39)
**Grade 5**				
	**Therapeutic hypothermia (n=45)**	**Controls (n=177, 843)**	**Crude mean difference (95% CI)**	**Adjusted mean difference (95% CI)**
Total z-score across 5 domains (SD)	-0.72 (1.7)	0 (1.0)	-0.72 (-1.02, -0.43)	-0.70 (-0.97, -0.42)
	**Therapeutic hypothermia (n=45)**	**Controls (n=177, 843)**	**OR (95% CI)**	**aOR (95% CI)**

Met National Minimum Standard n (%)	29 (64.4)	150, 605 (84.7)	0.33 (0.18, 0.60)	0.31 (0.16, 0.60)
	**No therapeutic hypothermia (n=29)**	**Controls (n=177, 843)**	**Crude mean difference (95% CI)**	**Adjusted mean difference (95% CI)**
Total z-score across 5 domains (SD)	-1.54 (1.9)	0 (1.0)	-1.54 (-1.91, -1.18)	-1.37 (-1.71, -1.028)
	**No therapeutic hypothermia (n=29)**	**Controls (n=177, 843)**	**OR (95% CI)**	**aOR (95% CI)**
Met National Minimum Standard n (%)	15 (51.7)	150, 605 (84.7)	0.19 (0.09, 0.40)	0.21 (0.09, 0.47)
**Grade 7**				
	**Therapeutic hypothermia (n=17)**	**Controls (n=75,163)**	**Crude mean difference (95% CI)**	**Adjusted mean difference (95% CI)**
Total z-score across 5 domains (SD)	-0.48 (1.5)	0 (1.0)	-0.48 (-0.95, -0.00)	-0.49 (-0.91, -0.03)
	**Therapeutic hypothermia (n=17)**	**Controls (n=75,163)**	**OR (95% CI)**	**aOR (95% CI)**
Met National Minimum Standard n (%)	14 (82.4)	59, 998 (79.8)	1.18 (0.34, 4.10)	1.08 (0.30, 3.92)
	**No therapeutic hypothermia (n=21)**	**Controls (n=75,163)**	**Crude mean difference (95% CI)**	**Adjusted mean difference (95% CI)**
Total z-score across 5 domains (SD)	-1.13 (1.9)	0 (1.0)	-1.13 (-1.56, -0.71)	-1.13 (-1.53, -0.72)
	**No therapeutic hypothermia (n=21)**	**Controls (n=75,163)**	**OR (95% CI)**	**aOR (95% CI)**
Met National Minimum Standard n (%)	13 (61.9)	59,998 (79.8)	0.41 (0.17, 0.99)	0.44 (0.17, 0.18)

Supplement 3: Growth modelling of academic score trajectories by HIE severity						
Parameters and growth predictors	Crude coefficient (SE)	95% CI	p-value	Adjusted coefficient (SE)	95% CI	p-value
Intercept (fixed difference)						
No HIE	Baseline					
Mild HIE	29.52	-5.74, 64.78	0.101	31.49	-2.85, 65.83	0.072
Moderate-severe HIE	-66.76	-88.41, -45.10	0.000	-57.16	-78.6, -35.72	0.000
Trajectory (gradient/ slope between grade 3-7)						
No HIE	30.96	30.86, 31.06	0.000	30.94	30.84, 31.03	0.000
Mild HIE	17.80	10.28, 25.22	0.000	17.4	9.94, 24.86	0.000
Moderate-severe HIE	28.47	23.97, 32.98	0.000	27.42	22.89, 31.94	0.000
Grades # HIE (gradient/ slope between grade 3 to 7 relative to controls)						
No HIE	Baseline					
Mild HIE	-13.16	-20.68, -5.63	0.001	-13.54	-21.00, -6.07	0.000
Moderate-severe HIE	-2.49	-6.99, 2.02	0.279	-3.52	-8.04, 1.01	0.128

Adjusted for: sex, indigenous status, parental education, ARIA (rurality score), socioeconomic status, smoking in pregnancy, birthweight Z score and week of gestation

Supplement 4: Growth modelling of academic score trajectories by HIE severity excluding those who did not receive therapeutic hypothermia						
Parameters and growth predictors	Crude coefficient (SE)	95% CI	p-value	Adjusted coefficient (SE)	95% CI	p-value
Intercept (fixed difference)						
No HIE	Baseline					
Mild HIE	38.9	11.3, 89.2	0.129	46.0	-3.09, 95.2	0.066
Moderate-severe HIE	-45.4	-73.0, -17.7	0.001	-37.4	-64.9, -10.0	0.008
Trajectory (gradient/ slope between grade 3-7)						
No HIE	31.0	30.9, 31.1	0.000	30.9	30.8, 31.0	0.000
Mild HIE	18.0	6.0, 30.0	0.003	17.9	6.1, 29.8	0.003
Moderate-severe HIE	27.2	20.9, 33.5	0.000	26.9	20.6, 33.1	0.000
Grades # HIE (gradient/ slope between grade 3 to 7 relative to controls)						
No HIE	Baseline					
Mild HIE	-13.0	-25.0, -1.0	0.034	-13.0	-24.8, -1.1	0.032
Moderate-severe HIE	-3.8	-10.0, 2.5	0.24	-4.0	-10.3, 2.2	0.206

Adjusted for: sex, indigenous status, parental education, ARIA (rurality score), socioeconomic status, smoking in pregnancy, birthweight Z score and week of gestation

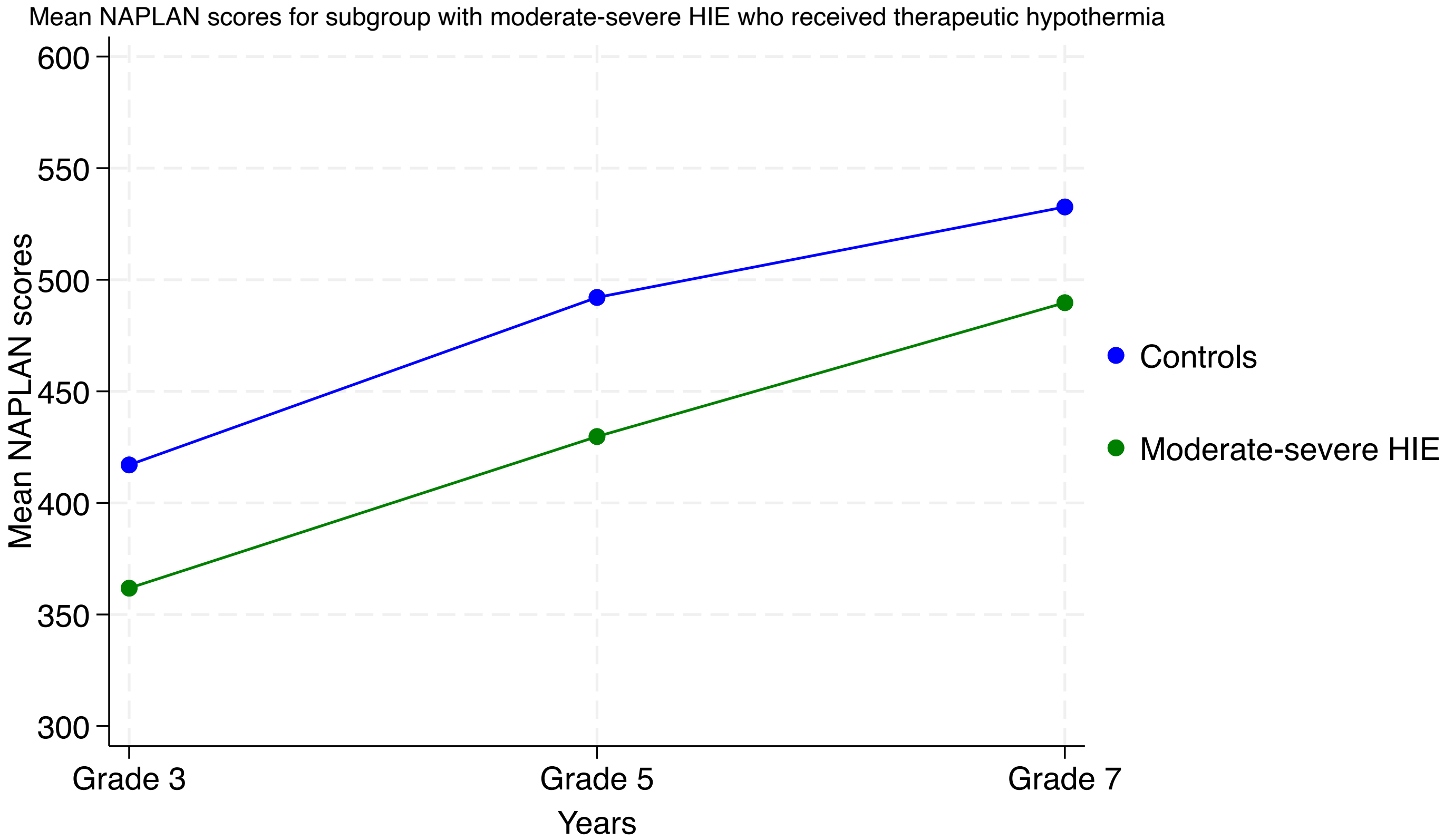

Supplement 5: Mean NAPLAN scores of infants with moderate-severe HIE who received therapeutic hypothermia relative to controls
